# Supraspinatus Tenotomy in Reverse Shoulder Arthroplasty for Fractures: A Comparative Cohort Study

**DOI:** 10.1177/21514593211019973

**Published:** 2021-07-07

**Authors:** Georg Siebenbürger, Evi Fleischhacker, Johannes Gleich, Tobias Helfen, Wolfgang Böcker, Ben Ockert

**Affiliations:** 1 Shoulder and Elbow Service, Department of General, Trauma and Reconstructive Surgery, Munich University Hospitals, Ludwig-Maximilians-University, Germany

**Keywords:** RSA, reverse total shoulder arthroplasty, proximal humeral fracture, fracture, shoulder, tenotomy, arthroplasty, humerus, hemiarthroplasty, supraspinatus tendon, rotator cuff

## Abstract

**Background:**

The aim of this study was to evaluate the functional and radiographic outcome in patients with reverse total shoulder arthroplasty (RSA) for displaced proximal humeral fractures (PHF) with or without tenotomy of the supraspinatus tendon.

**Methods:**

Between June 2011 and June 2018, 159 patients (age >65 years) with a displaced proximal humeral fracture underwent reverse total shoulder arthroplasty (Grammont design) in a single-center study and were longitudinally followed up. In all cases, the tuberosities were attached to the prosthesis in a standardized procedure. Functional outcome, range of motion as well as tuberosity integration, resorption and displacement were assessed at final follow-up. Outcomes were compared between patients that underwent RSA in combination with tenotomy of the supraspinatus (ST) and patients that underwent RSA without supraspinatus tenotomy (NT).

**Results:**

At a mean follow up of 22.2 ± 16.4 months 76 patients (mean age 77.1 ± 7.2 years, 83% women) could be evaluated (follow-up rate 47.8%). There were no statistically significant differences between the ST (n = 29) and NT groups (n = 47) in tuberosity integration, resorption </≥50%, or displacement (p = 0.99/0.31/0.7/0.99). Functional outcome was better in ST group (Constant score 76.2 ± 5.9 vs. 64.5 ± 12.8; p < 0.05) especially regarding mean active external rotation (>20°: 65.5% vs. 14.9%, p < 0.05) and active abduction (>120°: 89.7% vs. 21.3%, p < 0.05). Tuberosity integration (ST and NT together: n = 34) showed better functional results than resorption or displacement (p < 0.05).

**Conclusions:**

Tenotomy of the supraspinatus tendon in RSA for displaced PHF leads to similar radiographic results regarding tuberosity integration, resorption and displacement but better functional outcome with regard to range of motion.

**Level of Evidence:**

III

## Introduction

Fractures of the proximal humerus account for 5% of all fractures, with a high prevalence in elderly patients.^1,2^ Whereas non-displaced fractures can be treated conservatively, primary reverse total shoulder arthroplasty (RSA) may be considered as an alternative to fracture fixation for complex displaced proximal humeral fractures (PHF) in elderly patients with poor bone quality. Due to the increasing number of implanted RSA in recent years, this has overcome the number of implanted hemi-/anatomic arthroplasties. ^3,4^

As with anatomic shoulder arthroplasty for fractures, several studies have shown that fixing the tuberosities around the metaphysis of RSA leads to better function, especially external rotation and increases prosthetic stability.^5-9^ There are also studies, nevertheless, that have found no differences in functional results after refixation of the tuberosities and subsequent dislocation or resorption, compared to patients with healed tuberosities.^7,10,11^ A similar initial situation exists in patients with cuff arthropathy. Due to the degenerative changes, the SSP tendon is absent or defective. Despite this, these patients have a better outcome than those who had the RSA implanted because of a fracture and in whom the tuberosities have been refixed.^12-14^ Recent studies on RSA in proximal humerus fracture show improved stability of the tuberosity construct and no functional disadvantage after excision of the tendon. Furthermore, this allows for better intraoperative visualization of the glenoid, enabling even more precise implant positioning.^15,16^ However, there is only the article of Bonnevialle that investigated on the functional outcome of patients following RSA for fractures with tenotomy of the supraspinatus in comparison to the results without the tenotomy.^15^

Therefore, the aim of this study was to assess patients with proximal humerus fractures and after implantation of an RSA with or without tenotomy of the supraspinatus tendon with regard to radiological behavior of the tuberosity and functional outcomes. Our hypothesis was that tenotomy of the supraspinatus tendon would result in lower rates of tuberosity displacement because of the lack of tendon traction.

## Patients and Methods

### Study Design

Between June 2011 and June 2018, 159 patients with a displaced (>1 cm, 45° angulation) proximal humeral fracture and no evident cuff tear, were enrolled in this single center review board approved study after giving their informed consent. Evaluation of the rotator cuff was preoperatively evaluated by ultrasound and intraoperatively performed macroscopically by the surgeon for tears of the subscapularis, supra- and infraspinatus muscle. Fractures were classified according to Neer.^17,18^ In all cases, CT-scans were performed prior to surgical intervention. Minimum follow-up was 12 months. Exclusion criteria were open fractures, pathologic fractures resulting from metastatic or primary neoplasia, preoperative non- or malunion, revision surgeries, primary infections and preoperatively diagnosed neurological deficiency (lesions of the axillary or radial nerve, distinct dementia, condition after apoplectic insults and consecutive hemiparesis).

### Patient Demographics

Seventy-six patients were eligible to complete follow up and were assigned to group ST for Ssp tenotomy (n = 29) or group NT for Ssp preservation (n = 47). The mean age at surgery was 77.1 ± 7.2 years, 83% of patients were female. Mean age in group ST was 76 years, in group NT 78 years. The fracture pattern according to Neer classification was in total (n = 76): type III-2 3 patients (4%), type IV/V-3 14 patients (18%), type IV/V-4 19 patients (25%), and type VI 40 patient (53%). In group ST (n = 29): type III-2 2 patients (7%), type IV/V-3 5 patients (17%), type IV/V-4 3 patients (10%), and type VI 19 patient (66%). In group NT (n = 47): type III-2 1 patient (2%), type IV/V-3 9 patients (19%), type IV/V-4 17 patients (36%), and type VI 20 patient (43%). For detailed patient demographics see Table 1.

**Table 1.** Patients Demographics and Fracture Pattern in ST and NT Groups.

	Group ST	Group NT
Patients (n)	29	47
Mean Age (± STD)	76 ± 6.8	78 ± 7.3
Sex (n/% female)	26 / 79.3%	40 / 85.1%
Neer type III-2 (n/%)	2 / 7%	1 / 2%
Neer type IV/V-3 (n/%)	5 / 17%	9 / 19%
Neer type IV/V-4 (n/%)	3 / 10%	17 / 36%
Neer type VI (n/%)	19 / 66%	20 / 43%

### Surgical Procedure and Rehabilitation Protocol

All patients included were treated within 8 days of trauma by implantation of a primary reverse total shoulder arthroplasty. The procedure was performed in a beach chair position on a radiolucent table by 1 of 4 experienced trauma surgeons via a standardized deltopectoral approach. The same implant model was used in all cases (Aequalis Reversed FX; Wright-Tornier, Memphis, TN, USA). Standard 25- or 29-mm-diameter baseplates were implanted. Depending on the glenoid diameter measured intraoperatively resp. preoperatively, eccentric (+2 mm) 36- or 42-mm diameter glenospheres were subsequently placed. All stems had an inclination angle of 155° and were placed in 20° retroversion. Moreover they were cemented at the proper height to achieve tuberosity reconstruction, relatively to the medial calcar reference. Tuberosity fixation was performed in the Boileau technique with sutures and loops (Fiber Wire No. 5®; Arthrex Inc., Naples, FL, USA/Nice Loops; Wright-Tornier, Memphis, TN, USA).^19^ Beginning in August 2016 forward, tenotomy of the Ssp tendon was performed in all cases by resecting the tendinous part of the supraspinatus according to Bonnevialle et al and Miquel et al.^15,16^ Tenotomy of the Ssp tendon was executed using electrocautery and 2-5 mm proximal of the footprint to prevent bleeding of the highly vasculated bony insertion dependent on the size of the tuberosity fragment and fracture morphology over the whole footprint in anterior to posterior orientation. No other objective modifications in the surgical protocol were conducted. All patients received an abduction orthesis in the operations room [SAS multi comfort (15° abduction); Medi, Bayreuth, Germany]. The rehabilitation protocol allowed passive exercises on day 1 after surgery under supervision of a physiotherapist and unrestricted active range of motion after the third week.

### Clinical Assessment

The Constant Score (CS)^20,21^ [measurement of strength with a digital spring balance (Burg Wächter 76000 Tara PS®)]as well as the age and gender normalized CS(nCS) according to Katolik et al,^22^ the range of motion (ROM) with a goniometer and the pain with a visual analog scale (VAS) were assessed. External rotation was measured in axial plane with the arm by the patients’ side. The standardized follow-up included examination of the affected shoulder 6 weeks, 3, 6 and 12 months after surgery and at final follow-up. Statistical analysis was performed on the data at final follow-up.

### Radiographic Evaluation

**Figure 1.**
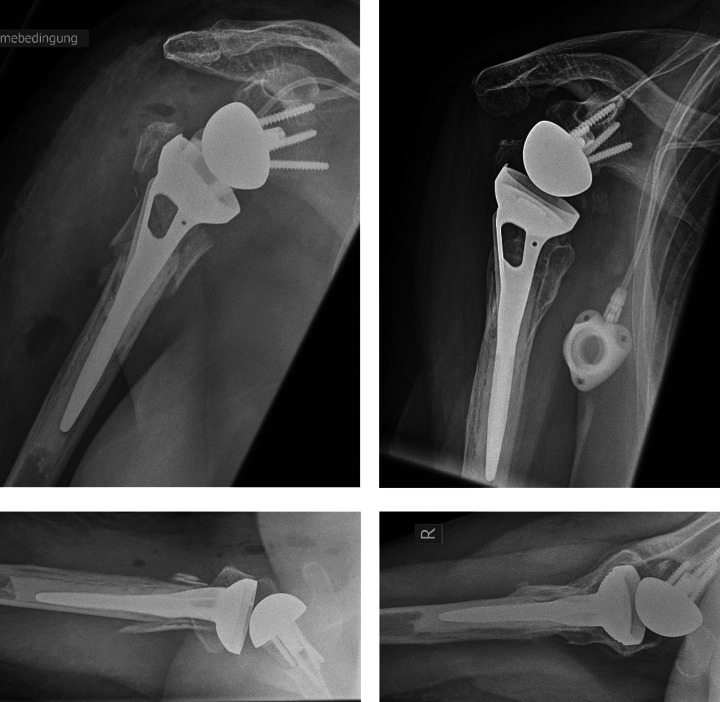
Postoperative plain radiographs in a.p. and axial radiographic view on the left side and secondary displacement of the greater tuberosity in RSA in a.p. and axial radiographic view on the right side.

In all patients true a.p., outlet view and axial radiographs were assessed the day after surgery and at every follow-up. Radiographs were evaluated for radiographic signs of tuberosity integration, resorption and displacement. Unless the tuberosities were dislocated, they were visible laterally on the stem and no more than 5 mm below the prosthetic head in diaphyseal continuity. When comparing the postoperative images and the radiographs at the follow-up visits, tubercular resorption was divided into less or more than 50%. >50%/<50% volume calculation of the tuberosity was performed using a math calculator for triangular areas. A representative example of tuberosity displacement is shown in Figure 1. Scapular notching and loosening were not observed in this study.

### Statistical Evaluation and Matched Pair Analysis

Continuous variables were described by means and standard deviation and were compared using Mann Whitney Test. Categorical variables were analyzed using Fisher’s Exact Test. The significance level for all tests was set at p < 0.05. Statistical analysis was performed using SPSS (IBM Corp. Released 2016. IBM SPSS Statistics for Windows, Version 24.0. Armonk, NY: IBM Corp.).

## Results

### Clinical Results

**Figure 2.**
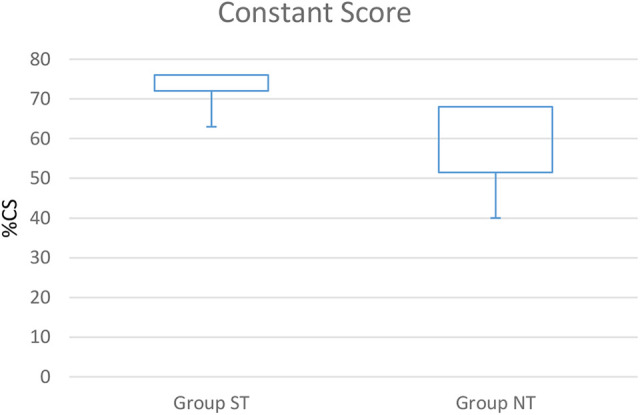
Clinical outcome box plot CS (p < 0.05).

**Figure 3.**
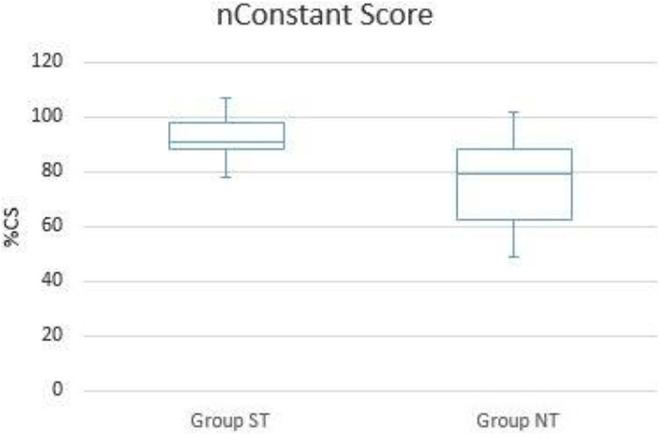
Clinical outcome box plot nCS (p < 0.05).

At mean follow-up of 22.2 ± 16.4 months, the mean Constant Score (CS)in group ST was 76.2 ± 5.9 points and the mean nCS 92.4 ± 7.2 points. In group NT, the mean CS was 64.5 ± 12.8 and the mean nCS 77.1 ± 15.7 see Table 2/Figures 2 and 3.

**Table 2.** Clinical Outcome With or Without Ssp Tenotomy (Mann-Whitney-U Test).

	Group ST	Group NT	p
Patients (n)	29	47	
Mean Age (±STD)	76 ± 6.8	78 ± 7.3	
CS ± STD	76.2 ± 5.9	64.5 ± 12.8	< 0.05
nCS ± STD	92.4 ± 7.2	77.1 ± 15.7	< 0.05

In a subgroup analysis of all cases with tuberosity integration (both in group ST and NT) (n = 34) the mean CS was 73.7 ± 8.1 points and in cases with tuberosity resorption or displacement 69.7 ± 12.1 points, see Table 3.

**Table 3.** Clinical Outcome With or Without Tuberosity Integration (Mann-Whitney-U Test).

	Complete integration of tuberosities	Tuberosity resorption or displacement	p
Patients (n)	34	42	
CS ± STD	73.7 ± 8.1	69.7 ± 12.1	< 0.05

Regarding ROM in group ST 26 patients (89.7%) achieved an active abduction of more than 120° whereas this was possible in only 10 patients (21.3%) in group NT. An active external rotation in the axial plane with the arm by the patients’ side was possible in 19 patients (65.5%) in group ST and in 7 patients (14.9%) in group NT, see Table 4.

**Table 4.** ROM With or Without Ssp Tenotomy (Fisher Exact Test); Comparison of Tuberosity Integration, Resorption and Displacement With or Without Ssp Tenotomy (Fisher Exact Test).

	Group ST	Group NT	p
Patients (n)	29	47	
Abduction >120° (n/%)	26/89.7%	10/21.3%	< 0.05
External rotation >20° (n/%)	19/65.5%	7/14.9%	< 0.05
Complete integration (n/%)	13/44.8%	21/44.7%	0.99
Resorption <50% (n/%)	11/37.9	18/38.3%	0.99
Resorption >50% (n/%)	2/7%	6/12.8%	0.70
Displacement (n/%)	3/10.3%	2/4.2%	0.31

We did not observe any major complications (soft tissue infection, loosening, breakage, secondary bleeding) nor was any revision surgery needed.

### Radiographic Results

**Figure 4.**
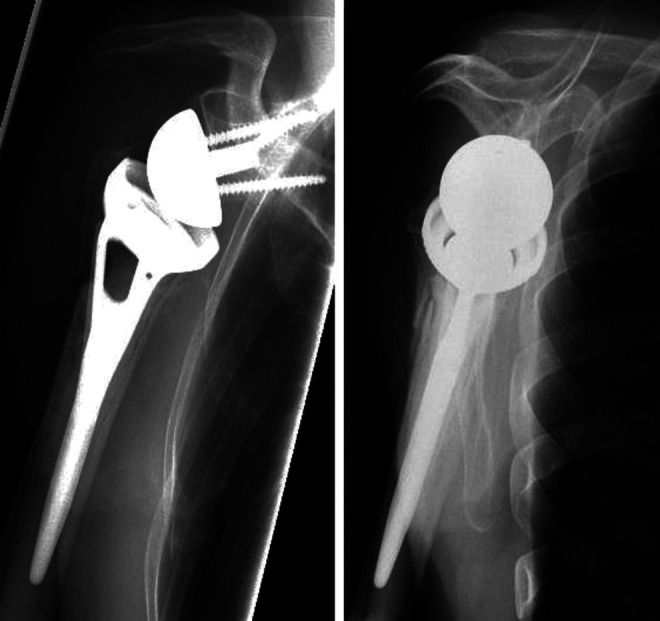
Complete integration of the tuberosities in RSA in a.p. and y-view radiographic view.

In group ST (n = 29) complete tuberosity integration was seen in 13 cases (44.8%) [see Figure 4], resorption <50% was seen in 11 cases (37.8%), resorption over 50% was seen in 2 cases (7%) and displacement in 3 cases (10.3%) [see Figure 1]. In group NT (n = 47) complete tuberosity integration was seen in 21 cases (44.7%), partial resorption <50% was seen in 18 cases (38.3%), resorption over 50% was seen in 6 cases (12.8%) and displacement in 2 cases (4.2%). There was no statistically significant difference comparing both groups in regard of tuberosity integration, resorption or displacement whether or not a Ssp tenotomy was made, see Table 4. We did not observe any cases of notching.

## Discussion

This study shows no significant radiographic difference regarding tuberosity integration, resorption or displacement following primary RSA for displaced PHF with or without supraspinatus tenotomy. Nevertheless, we observed better functional outcome following tenotomy of the Ssp tendon in RSA for PHFs especially for active abduction >120° and active external rotation >20°. Both, a higher abduction as well as external rotation may be advantageous for elderly patients to stay independent in their daily activities.

From a technical surgical view, a better visibility of the glenoid cavity is possible after tenotomy of the Ssp tendon, which is advantageous for implantation of the glenoid component and tuberosity fixation to the metaphysis. However, we did not observe differences in terms of implant position. Nevertheless, better visualization and easier fixation of the tuberosity may lead to shorter operative time, which we did not analyze in this study, and thus to less cardiopulmonary burden in geriatric patients. Another aspect is that, due to the RSA design, an existing Ssp is not necessary for biomechanical function but, on the other hand, can cause cranial soft tissue impingement and is then responsible for persistent discomfort.^15^

Despite we saw no higher rate of healing tuberosities in the Ssp tenotomy group and did not use glenospheres with a wider diameter, this might be biomechanically explained by a better motion of the “socket” around the “ball” in the RSA design preventing from soft tissue impingement and arthrofibrosis. In addition, better biomechanical lever of the deltoid muscle because of a distalized center of rotation may predict better abduction and forward flexion. The centralization of the prosthesis in motion because of a more stable tuberosity reconstruction and optimized muscular power because of higher tension of the infraspinatus, teres minor and subscapularis tendon may explain the better external rotation in patients that underwent Ssp tenotomy.^16^

Based on our hypothesis, we expected a higher rate of tuberosity integration and, conversely, a lower rate of displacement with Ssp tenotomy because of the lack of force transmission from the supraspinatus muscle to the major tubercle.

From the results of our study, however, there was no difference in the rate of tuberosity integration compared to patients in which tenotomy was not performed. One potential reason may be a higher rate of tuberosity resorption due to the phenomenon known as Wolffs’ law with lacking traction forces on the major tubercle.^16^ However, we did not observe significant differences in the radiographic behavior of the tuberosities between the 2 groups. In turn, functional outcomes with regard to integration of the tuberosities showed similar findings to other studies, suggesting a favorable outcome with integrated tuberosities than without, independently of supraspinatus tenotomy. Some past work showed that patients whose tuberosities had been refixed and healed had better functional outcomes than those without tuberosity repair.^5,15,23^ For example Bonnevialle et al.^15^ observed comparable results regarding tuberosity integration and displacement. Similar to our findings they showed that tenotomy of the Ssp tendon leads to better external rotation in RSA for fractures. These results were confirmed by 2 systematic reviews by Anakwenze et al and Jain et al. ^5,23^ The biomechanical work of Miquel et al provides further evidence that a more stable tuberosity construct improves external rotation through the teres minor and infraspinatus.^16^

We did not observe major complications in any group, therefore the argument Ssp excision may increase the “dead space” and consequently poses a potential risk for secondary postoperative hematoma and infection is not supported by our results. In a previous study, Florschutz et al found similar results when comparing hemiarthroplasty and RSA.^24^

The results of this study have to be seen in the light of its limitations. Its retrospective design and a mean follow-up period of 2 years in total with a minimum follow-up period of 12 months may be considered too short to assess on radiographic behavior of the tuberosities and the final clinical outcome. However, this is the first study to compare the functional outcomes of additive Ssp tenotomy in RSA for fractures. Furthermore the follow-up parameters are similar to other studies examining an orthogeriatric patient population.

Second, the decision for Ssp tenotomy was made from an otherwise unspecific time point forward. Though there were no other objective modifications in the surgical protocol, one may argue, that tuberosity fixation may be performed more precisely from this time ongoing. However, the tuberosities were fixed meticulously in all patients of this study and differences may be minimal. Third, radiological assessment of tuberosities was made with 3 conventional radiographic planes only and therefore some resorptions may be overestimated or underestimated. CAT scans may have provided a more reliable assessment of radiographic complications but were not authorized by the ethical review board.

## Conclusion

Tenotomy of the supraspinatus tendon in RSA for PHF leads to similar results regarding tuberosity integration, resorption and displacement but better functional outcome, especially external rotation and abduction. Therefore, tenotomy of the supraspinatus tendon may be suggested in cases of primary RSA for PHF in elderly patients.
